# The Beat

**Published:** 2011-05

**Authors:** Erin E. Dooley

## NTP Issues Draft Report on Aloe Vera

Many aloe beverages on the market today are made with decolorized aloe, in which charcoal filtration removes biochemical components such as aloin, a suspected carcinogen and one of the anthraquinones thought to give the plant its laxative properties. However, a lack of federal labeling requirements means consumers cannot be sure whether or how much aloin is present in any given product. In response to health concerns about aloin and other aloe constituents, the National Toxicology Program (NTP) recently completed a 2-year rodent assay of nondecolorized extract of aloe vera (derived from the green part of the leaf as opposed to the clear gel inside).^1^ The program’s draft report, issued in April, concludes that rats given water containing 60 ppm aloin—6 times the amount allowed in orally ingested products under self-imposed industry standards—39% of females and 74% of males developed malignant or benign intestinal tumors. The human health implications of these findings are unclear. A final NTP report is expected in 2012.

**Figure:**
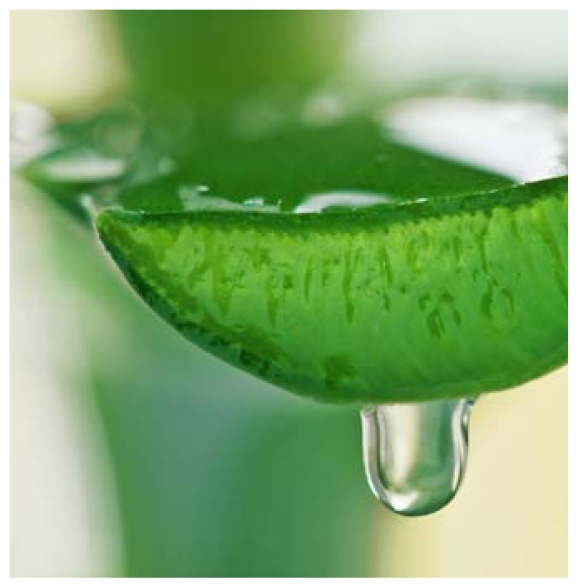
Aloe vera juice is marketed as a laxative and digestive tonic.

## OSHA Issues Alert on Formaldehyde in Hair-Straightening Products

As a result of concerns from salon employees, federal and state officials are investigating worker exposures to formaldehyde during use of hair-straightening products, some of which contain the chemical without listing it on the label. One investigation documented air levels of formaldehyde in excess of OSHA limits for salons even though the product used was labeled “formaldehyde-free.” In April 2011 OSHA issued a hazard alert to warn salon owners and workers about the potential health effects of formaldehyde, ways to determine if products contain the chemical, and steps to reduce exposure.^2^ Formaldehyde, a known human carcinogen, can also irritate the nose and eyes and cause adverse allergic and neurologic effects.^3^

## Global Study Finds cVMS Widely Distributed in Air

cVMS, or cyclic volatile methyl siloxanes, are high-production-volume chemicals used in personal care products to make them feel silky and to help them dry quickly. A new study of cVMS in ambient air found the compounds clustered in varying distributions at each of 20 sites around the world, including 5 Arctic sites.^4^ The D5 and D6 species of cVMA concentrated in the urban sites studied, whereas the D3 and D4 species were especially elevated along the U.S. West Coast. D5 and D6 are the cVMS used most commonly in personal care products, while D3 and D4 are thought to be associated with industries that produce silicone polymers. Currently there are no restrictions on any use of cVMS, but regulators in a number of countries are paying more attention to these compounds because of evidence they may be persistent, bioaccumulative, and toxic.

**Figure:**
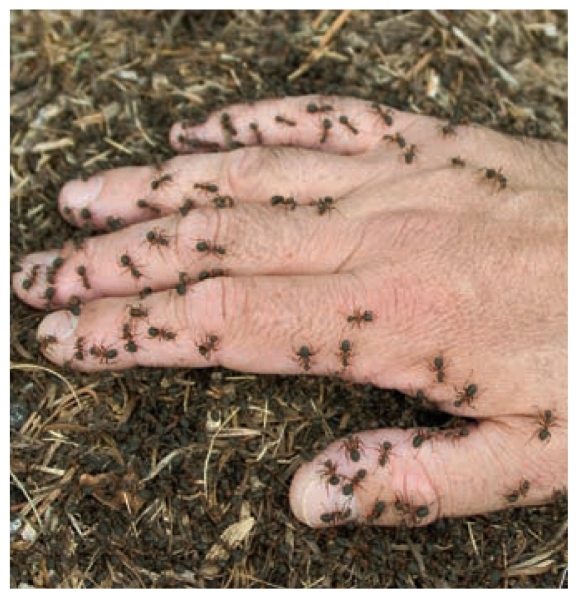
Ant poison containing banned mirex was being sold online.

## EPA Warns of Illegal Pesticide Sales Online

In April 2011 the U.S. EPA announced it had warned almost 3,000 customers across the country about Fast Ant Bait products they had purchased online.^5^ The products contain mirex, a pesticide banned since 1978 because of its adverse liver, skin, reproductive, and neurologic effects.^6^ When EPA officials discovered the products were being sold online, they ordered the online payment company to cease processing orders for the Chinese-made and -distributed products. The EPA notified U.S. customers about the health risks posed by the products and how to properly clean up and dispose of them. Canada’s Pest Management Regulatory Agency has notified customers in that country as well.
